# Magnetic Resonance Imaging Sequence Identification Using a Metadata Learning Approach

**DOI:** 10.3389/fninf.2021.622951

**Published:** 2021-11-17

**Authors:** Shuai Liang, Derek Beaton, Stephen R. Arnott, Tom Gee, Mojdeh Zamyadi, Robert Bartha, Sean Symons, Glenda M. MacQueen, Stefanie Hassel, Jason P. Lerch, Evdokia Anagnostou, Raymond W. Lam, Benicio N. Frey, Roumen Milev, Daniel J. Müller, Sidney H. Kennedy, Christopher J. M. Scott, The ONDRI Investigators, Angela Troyer, Stephen C. Strother

**Affiliations:** ^1^Rotman Research Institute, Baycrest Health Center, Toronto, ON, Canada; ^2^Indoc Research, Toronto, ON, Canada; ^3^Robarts Research Institute, Western University, London, ON, Canada; ^4^Department of Medical Imaging, Sunnybrook Health Sciences Centre, Toronto, ON, Canada; ^5^Department of Psychiatry, Cumming School of Medicine, University of Calgary, Calgary, AB, Canada; ^6^Mouse Imaging Centre, Hospital for Sick Children, Toronto, ON, Canada; ^7^Wellcome Centre for Integrative Neuroimaging, FMRIB, Nuffield Department of Clinical Neurosciences, University of Oxford, Oxford, United Kingdom; ^8^Bloorview Research Institute, Holland Bloorview Kids Rehabilitation Hospital, Toronto, ON, Canada; ^9^Department of Pediatrics, University of Toronto, Toronto, ON, Canada; ^10^Department of Psychiatry, University of British Columbia, Vancouver, BC, Canada; ^11^Department of Psychiatry and Behavioral Neurosciences, McMaster University, Hamilton, ON, Canada; ^12^Mood Disorders Program, St. Joseph’s Healthcare, Hamilton, ON, Canada; ^13^Departments of Psychiatry and Psychology, Providence Care Hospital, Queen’s University, Kingston, ON, Canada; ^14^Molecular Brain Science, Centre for Addiction and Mental Health, Campbell Family Mental Health Research Institute, Toronto, ON, Canada; ^15^Department of Psychiatry, University of Toronto, Toronto, ON, Canada; ^16^Department of Psychiatry, Krembil Research Centre, University Health Network, Toronto, ON, Canada; ^17^Department of Psychiatry, St. Michael’s Hospital, University of Toronto, Toronto, ON, Canada; ^18^Keenan Research Centre for Biomedical Science, St. Michael’s Hospital, Li Ka Shing Knowledge Institute, Toronto, ON, Canada; ^19^L.C. Campbell Cognitive Neurology Research Unit, Toronto, ON, Canada; ^20^Heart & Stroke Foundation Centre for Stroke Recovery, Toronto, ON, Canada; ^21^Sunnybrook Health Sciences Centre, Brain Sciences Research Program, Sunnybrook Research Institute, Toronto, ON, Canada; ^22^Department of Medical Biophysics, University of Toronto, Toronto, ON, Canada

**Keywords:** health data, MRI sequence naming standardization, data share and exchange, machine learning, metadata learning, AI-assisted data management

## Abstract

Despite the wide application of the magnetic resonance imaging (MRI) technique, there are no widely used standards on naming and describing MRI sequences. The absence of consistent naming conventions presents a major challenge in automating image processing since most MRI software require *a priori* knowledge of the type of the MRI sequences to be processed. This issue becomes increasingly critical with the current efforts toward open-sharing of MRI data in the neuroscience community. This manuscript reports an MRI sequence detection method using imaging metadata and a supervised machine learning technique. Three datasets from the Brain Center for Ontario Data Exploration (Brain-CODE) data platform, each involving MRI data from multiple research institutes, are used to build and test our model. The preliminary results show that a random forest model can be trained to accurately identify MRI sequence types, and to recognize MRI scans that do not belong to any of the known sequence types. Therefore the proposed approach can be used to automate processing of MRI data that involves a large number of variations in sequence names, and to help standardize sequence naming in ongoing data collections. This study highlights the potential of the machine learning approaches in helping manage health data.

## Introduction

Magnetic resonance imaging (MRI), as a non-invasive technology that can provide detailed images of organs and tissues in the body, has been routinely used in early detection and diagnosis of various cerebral and cardiovascular diseases (Teipel et al., 2013; Lemaître et al., 2015; Zhou et al., 2015; Abbasi and Tajeripour, 2017). An MRI session is typically obtained by a combination of different radiofrequency pulses and field gradients’ settings from an MRI scanner, which results in multiple MRI sequences, or scans, each providing a different perspective of the examined tissues (Collins, 2016; Calle and Navarro, 2018). Despite the wide applications of the MRI technique and efforts to standardize their reporting (Manfredi et al., 2018), there are no widely used standards on naming and describing the MRI sequences. Different naming conventions have been used in different institutes and/or research groups, and the naming conventions could also be changed over time within the same institutes. Moreover, software upgrades to the scanner itself may accidentally necessitate changes to the naming conventions, even mid-study. On the other hand, most MRI data processing and pre-processing software packages require *a priori* knowledge of the type of the MRI sequences to be processed, where the file/folder names are typically used to identify a particular scan type from an MRI session. For example, one needs to identify the T1-weighted images and pass the file/folder names to FreeSurfer’s recon-all command^1^. Even small variations, such as extra spaces or mixed usage of hyphens and dashes, in sequence names, can cause problems to computer software programs. Therefore, the absence of consistent naming conventions presents a major challenge in automating image processing and pre-processing procedures. This issue becomes increasingly critical giving the current efforts toward open sharing of MRI data in the neuroscience community (Laird et al., 2005; Teeters et al., 2008; Yarkoni et al., 2011; Hall et al., 2012; Poldrack et al., 2013; Ferguson et al., 2014; Vaccarino et al., 2018), and the wide interests in performing meta-analyses of neuroimaging studies (Müller et al., 2018). The rapid growth of the volume and heterogeneity of the data makes it unrealistic to process without an automated approach.

One approach toward fully automatic processing of large amounts of heterogeneous MRI data is to develop and deploy standardized ontologies and protocols for data capture and curation. There have been a number of efforts toward this direction (Teeters et al., 2008; Poldrack et al., 2013; Van Horn and Gazzaniga, 2013; Rotenberg et al., 2018; Vaccarino et al., 2018). The OpenfMRI project (Poldrack et al., 2013) developed specific file naming schemes for organizing task based functional MRI data. At Brain-CODE, we have developed various quality assurance and quality control (QA/QC) pipelines to help enforce standard naming of MRI data from different study programs and research institutes (Vaccarino et al., 2018). The Brain Imaging Data Structure (BIDS) standard was also proposed to organize and describe neuroimaging data where the specification covers MRI sequences from most common experiments (Gorgolewski et al., 2016; Niso et al., 2018). The success of this approach, however, requires a global coordination to develop effective and practical standards, and requires early planning and consistent quality control through data acquisition and sharing processes. While this approach is promising for collecting prospective data, the challenge for identifying and reorganizing existing data still remains. Previously, aligning non-standardized names involved a lot of manual effort, which represents a significant portion of work in data platform development (Poldrack et al., 2013; Van Horn and Gazzaniga, 2013). To help fulfill the needs for automating data processing, the proposed work adopts a machine learning approach to identify the type of MRI sequences using imaging metadata.

The neuroscience community has used two principal data formats for MRI data storage. The Digital Imaging and Communications in Medicine (DICOM) format was proposed in the 1980s to help manage medical image information, and it has been adopted widely by medical imaging equipment vendors and healthcare organizations (Mildenberger et al., 2002). The DICOM standard is very comprehensive and flexible. It uses a tag-based format to encode information about the patient/participant, the device, and imaging sequence specifics, in the imaging file headers. In recent years, a neuroimaging informatics technology initiative (NIfTI) format has also been widely adopted^2^. The NIfTI standard specifies a relatively limited space (348-bytes) for data headers. Although it has been proposed that more detailed metadata regarding the imaging sequences should be provided in conjunction to the image data in NIfTI format (Turner and Laird, 2012; Poldrack et al., 2013; Gorgolewski et al., 2016), there is no widely used systematic framework for generating imaging metadata when performing DICOM to NIfTI conversions. In addition, the DICOM to NIfTI conversion may need different procedures for different imaging sequences (Li et al., 2016). Therefore the DICOM file format is more suitable for the purpose of identifying MRI sequences.

By the DICOM standard, the embedded metadata in DICOM headers should, in theory, be able to identify the type of an MRI sequence^3^. HeuDiConv^4^ and ReproIn^5^ are two recent software projects conducted to facilitate identifying MRI sequences using built-in or user-provided criteria. In practice, however, some DICOM headers could be left blank or even be filled incorrectly. It has previously been reported that over 15% of images contained false headers that can cause incorrect categorization of sequences (Gueld et al., 2002). This is also in line with our experience with Brain-CODE data platform (Vaccarino et al., 2018). A check of data collected by two Brain-CODE study programs indicated that nearly half of the sequences collected missed the DICOM header (*0018*,*0024*) *Sequence Name*, which represents the manufacturer’s designation of the sequence names^6^. This makes it rather challenging to set up predetermined criteria to identify MRI sequence types. In addition, some sequences like *MR Localizer* may have the same values in some relevant DICOM headers as other sequences, which can cause incorrect labeling for automated sequence identification. On the other hand, with the rich metadata encoded in DICOM headers, there could be multiple routes to determine the type of an MRI sequence. Even with a subset of the DICOM headers missing and/or being incorrect, it is still possible to identify the type of an MRI sequence by other existing DICOM headers. This is a classic machine learning classification problem where the DICOM header fields provide a rich set of features to classify the MRI scans. In the proposed work, we adopted the random forest technique, which is a supervised machine learning algorithm that leverages a large number of relatively uncorrelated decision paths to determine its prediction (Breiman, 2001), to predict the MRI sequence types. The random forest algorithm has been shown to have strong fault tolerance and high prediction accuracy for prediction data with distorted information (Kaur and Malhotra, 2008).

In this manuscript, we report our first attempt to use a random forest model and imaging sequence metadata to identify MRI sequence types. Three datasets from the Brain-CODE data platform, each involving MRI data with nominally aligned dataset-specific MRI sequences from multiple scanners/institutes, are used to build and test our model. The datasets are provided by three independent disease programs funded by the Ontario Brain Institute: the Ontario Neurodegeneration Disease Research Initiative (ONDRI), the Canadian Biomarker Integration Network in Depression (CAN-BIND), and the Province of Ontario Neurodevelopmental Disorders Network (POND). The preliminary results show that a random forest-based model can be trained to accurately identify MRI sequence types, and to flag MRI sequence types that are unknown to the model.

## Materials and Methods

### Data Preparation

#### Dataset Description

We used MRI data from the three independent research programs, ONDRI, CAN-BIND, and POND, from Brain-CODE. These data were scanned at multiple research institutes and hospitals across Canada, and were then uploaded to the Stroke Patient Recovery Database (SPReD)^7^, an XNAT (Marcus et al., 2007) based imaging server in the Brain-CODE platform. There were a total of 1,853 subjects, and 3,642 imaging sessions collected by the end of the year 2019, when the metadata was prepared for the present study. The counts of research sites, subjects, sessions, and scans are summarized in Table 1.

**TABLE 1:** A summary of the datasets used in this study.

	**ONDRI**	**CAN-BIND**	**POND**	Total*
Sites	14	10	5	29
Subjects	542	621	690	1,853
Sessions	1,475	1,369	798	3,642
Scans	12,326	12,958	9,407	34,691

*^∗^Note there might be overlap in research sites from the three research programs. We did not try to remove the duplicates when counting the sites in the present study.*

#### Data Labeling

Tremendous efforts have been made to standardize the naming of the data collected and stored on Brain-CODE. For MRI data, we have developed pipelines to check the scan name and parameters, aligning different naming conventions from different research institutes into Brain-CODE standard. For the present work, other major challenges are posed by scans that were not required by the study programs but were uploaded to the platform as ancillary data, as well as retrospective data acquired before corresponding naming standards had been developed and implemented. This represents over 30% of the total sequences used in this study, most of which have been labeled manually. There are 2,350 scans (261 from CAN-BIND and 2,089 from POND project), most derived images, out of a total of 34,691 scans that we were not able to identify. These scans were excluded from the current study. Table 2 summarizes the number of scans of each MRI sequence type used in the present study.

**TABLE 2:** A summary of the magnetic resonance imaging (MRI) sequences for the machine learning model training and testing*^1^.

**Sequence description**	**MRI sequence**	**Count**
T1 weighted images	3DT1	3,807
Fluid attenuated inversion recovery images with T2 contribution	2D FLAIR	1,458
Proton density and T2 weighted images	PD/T2	2,384
T2star weighted images	T2-star	1,773
Diffusion tensor images	DTI	9,385
Functional MRI images	fMRI	7,482
Multiple image types*^2^	junk	4,589
Arterial spin labeling perfusion images	ASL	87
Field mapping data	Field Map	1,376

^*1^Out of 34,691 sequences listed in Table 1, there were 2,350 scans (261 from CAN-BIND and 2,089 from POND project), most derived images, that we were not able to identify and label. These sequences are excluded from the current study. ^*2^This “junk” category includes sequences of Localizer, AAHead_Scout, Calibration, etc., that are not typically used for subsequent research.

### Machine Learning Procedures

#### Feature Selection

A list of sequence metadata, including sequence specific headers, timing parameters, spatial and contrast properties, and hardware manufacturers, of DICOM images are selected as the features for the present machine learning study, as shown in Table 3. The selection of the features is primarily guided by the neuroscience domain knowledge from the authors, since the relationships between the various image metadata are better understood by domain experts, rather than the machine learning algorithms. In comparison with data-driven feature selection, domain knowledge guided feature selection can help prevent the problem of overfitting where the constructed models may not reflect the true relationships in the data set (Groves, 2013). The selected list of features has also been validated by the feature importance extracted from the constructed random forest model (see section “Feature Importance”).

**TABLE 3:** A list of the MRI sequence metadata used as features in the current machine learning model.

	**Sequence metadata**	**DICOM headers**
1	Scanning sequence	(0018,0020)
2	Sequence variant	(0018,0021)
3	Scan options	(0018,0022)
4	MR acquisition type	(0018,0023)
5	Sequence name	(0018,0024)
6	Image type	(0008,0008)
7	Repetition time	(0018,0080)
8	Echo time	(0018,0081)
9	Inversion time	(0018,0082)
10	Flip angle	(0018,1314)
11	Pixel bandwidth	(0018,0095)
12	Image orientation	(0020,0037)
13	Diffusion *b*-values*^1^	
14	FOV X	
15	FOV Y	
16	MultiEcho_TE1	
17	MultiEcho_TE2	
18	Scanner manufacturer	(0008,0070)

*These metadata include sequence specific tags (1–6), timing parameters (7–9, 16, 17), etc., that are encoded in DICOM headers, and some computed properties (14, 15). The corresponding DICOM headers are also presented in the table when available.*

^*1^Diffusion *b*-values are encoded in different DICOM headers depending on the scanner manufacturer. For GE, Philips, and Siemens scanners, this attribute is encoded in (0043,1039), (2001,1003), and (0019,100c), respectively.

A few criteria that we used for selecting the features listed in Table 3 are as follows.

(1) The features need to be sequence relevant.
(2) The features need to be machine generated or programmed properties. Any notes or comments made by humans, including DICOM header (0008,103E), which is commonly used for manually identifying sequence types, are not used in this study.
(3) The features have to exist in the majority of the scans. For example, although the 3rd and 4th gradient echoes can be useful in identifying relevant sequences, they only exist in a very small fraction of the scans. Therefore, only the first two gradient echoes are selected in this study.

All the selected features are retrieved from the SPReD platform through a programming interface provided by XNAT (Marcus et al., 2007). We note all these metadata represent the properties of the image itself and they can also be obtained from most other DICOM tools.

#### Feature Engineering

The selected features include metadata in numeric format (mainly MRI timing parameters) and in string format. We used the following feature engineering approaches to handle missing data, empty fields, and categorical features.

(1) Missing values are replaced with 1,000,000 for both categorical and numerical features, where it represents infinity in the latter case.
(2) Empty values are simply filled with the string “empty.” Here we need to differentiate between empty and missing values where empty values sometimes could mean a header does not have the corresponding value in some scans.
(3) All categorical features are firstly transformed into numerical values that represent the indices of each label in the category. One-hot encoding approach is used to map the categorical features to binary vectors (Harris and Harris, 2012). This approach checks each feature and identifies the total number of distinct values of that feature. If a feature has *n* distinct values, it will transform the feature into *n* columns where each column contains 0 or 1 indicating the absence or presence of the specific value. The transformed binary vector is then ready to fit and train the random forest model.

#### Hyperparameter Tuning

The random forest algorithm has several tunable hyperparameters such as the number of decision trees (*numTrees*), the maximum depth of each tree (*numDepths*), the maximum number of bins to discretize continuous features (*numBins*), and an impurity measure used to decide the optimal split from a root node (Breiman, 2001; Karalis, 2020). To optimize the performance of the random forest algorithm, we used a cross-validation method to tune three most relevant hyperparameters, *numTrees* (2–64), *numDepths* (2–24), and *numBins* (2–32). Other hyperparameters used default values from the implementation of machine learning library MLlib Spark 2.3.1 (Meng et al., 2016; Zaharia et al., 2016). An exhaustive grid search has been performed with a step size of 2 for each of the three hyperparameters, with a randomly selected 50 scans of each sequence type in Table 2 as the training dataset and the rest scans as the testing dataset. In other words, the training dataset includes 450 scans and the testing dataset includes 29,891 scans. We used the relatively small size of the training data because the prediction accuracy is too high to reflect the impact of the hyperparameters when the size of the training data reaches only ∼100 scans from each sequence type, as will be shown below.

**FIGURE 1:**
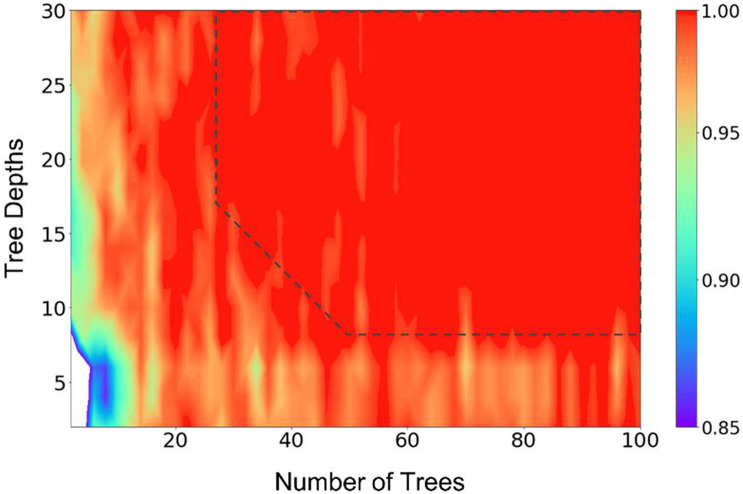
Results of a grid search of the number of trees and tree depths of the random forest model, illustrating the hyperparameter tuning processes. The number of bins were fixed at 32 in the calculations. The prediction accuracy was represented by the scale of the colors showing on the right side. The dashed line (drawn manually to serve as a guide to the eye) represents the approximate point where the prediction accuracies plateaued.

The cross-validation results show that the model can get a very good accuracy with *numTrees* = 40, *numDepths* = 8, and *numBins* = 16. Figure 1 presents the result of a grid search of numTrees and numDepths with numBins = 32, where we can see that the accuracy of the model reached a plateau with roughly *numTrees* = 32 and *numDepths* = 8, or *numTrees* = 24, and *numDepths* = 16. In the following model training and testing processes, we used *numTrees* = 100, *numDepths* = 16, and *numBins* = 32. Note the two latter hyperparameters are the maximum values that can be used in the model. We increased the *numTrees* to 100 since a higher number of trees can increase the accuracy of the prediction and make the predictions more stable but will not cause overfit the model.

#### Model Training and Testing

In the present work, we chose the sequences types 1–7 (Table 2) to build and test the random forest model, and used sequences 8 and 9 to test if the trained model can flag the sequences that are unknown to it. The latter test is critical in practical applications of the proposed method since sequences that do not exist in a training dataset are well expected.

Different sizes of training dataset, from 20 scans from each sequence, to 1,200 scans from each sequence, were used to build and test the model, while the rest of the scans were used as the testing dataset. For example, for a training size of 20, the training dataset includes 20 3DT1 scans, 20 2D FLAIR scans, and so on, while the testing dataset includes 3,807 – 20 = 3,787 3DT1 scans (since there are 3,807 3DT1 scans in total, see Table 2), 1,458 – 20 = 1,438 2D FLAIR scans, and so on. Such disproportionated stratified sampling ensures an adequate number of each sequence type in the training dataset, since the collected dataset is imbalanced. Twenty independent runs were performed on each size of training dataset, in order to estimate the variations of the prediction accuracies. With the manually selected list of features (section “Feature Selection”), and the hyperparameters determined using cross-validation method (section “Hyperparameter Tuning”), the trained model could consistently predict the sequence type with very good accuracy. Therefore, no additional optimization of the features or hyperparameters was performed in the work.

We then used a model that was built from 1,200 scans from each sequence type 1–7 to predict the scans of sequence types 8 and 9. The purpose of this step was to test whether the model is able to recognize scans that do not belong to any of the known sequence types. We calculated the prediction confidence of the scans. The prediction confidence, also known as prediction probabilities, is the probability of an observation belonging to the most likely class predicted by the model. We found that the prediction confidences of these sequences are significantly lower than that of predictions of known sequences. This suggested that the prediction confidence could be used to flag unknown scans in practical applications.

All the computations were performed on an in-house Spark cluster using Spark 2.3.1 (Zaharia et al., 2016). We note that the use of the Spark cluster is not essential for performing the present study, as a typical machine learning model training and testing process with the current dataset takes only about 3.5 mins on an average personal computer (4 Cores and 8 GB of RAM).

## Results

### Classification Accuracy

**FIGURE 2:**
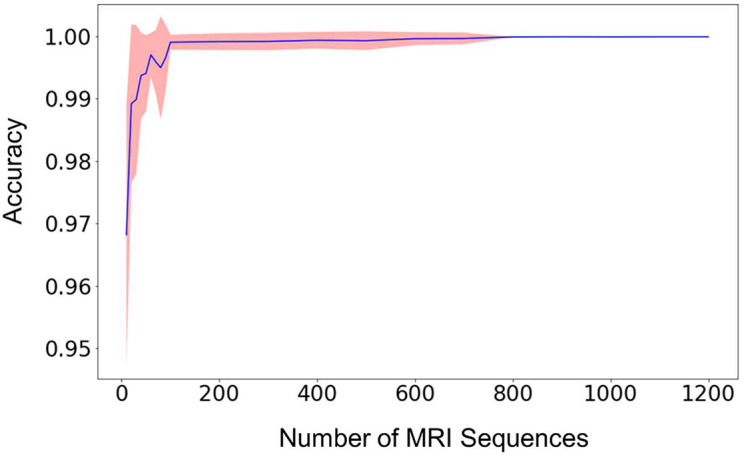
The mean prediction accuracy (blue line) and standard deviation (shaded area around the blue line) of the random forest model built from different sizes of training datasets. The prediction accuracy is defined as the fraction of the testing scans that were classified correctly. The X-axis (i.e., Size of MRI Sequences) represents the number of scans from each type of sequence 1–7 in Table 2, that are used in the training of the random forest model. The standard deviations are calculated from 20 independent computations. When the size reaches approximately 800, the standard deviation is smaller than the width of the line in the figure, and the prediction accuracy is consistently larger than 0.999.

The relationship between the classification accuracy, i.e., the fraction of correct predictions, of the testing dataset and the size of the training dataset was shown in Figure 2. For each size of the training dataset, 20 runs were performed independently to assess the reproducibility of the machine learning procedure and the variations of the prediction accuracy. From Figure 2, we can see that the average accuracy grew rapidly with the increase of the size of the training dataset. The standard deviation of the accuracies also decreased rapidly in the meantime, until the value was too small to be noticed from the plot.

When the number of the training sequences reached about 800 for each type of the selected MRI sequences, the classification accuracies were steadily over 0.999. In other words, for every 10,000 sequences, less than 10 scans were predicted incorrectly. Most of the incorrectly labeled scans are from category 7, “junk” type. This is because the category contains several sequence types (Localizer, AAHead_Scout, Calibration, etc.) and the number of the scans included in the training dataset represents the total of all these sequence types, so the low performance is expected. For example, when including 1,200 scans of each sequence type in the training dataset, 36 scans were incorrectly labeled out of 442326 sequence predictions (99.99% accuracy). Out of the 36 scans, 34 of them are from the category 7, “junk”. The other two incorrectly labeled scans are a 2D Flair scan and a PD/T2 scan. In other words, a good prediction accuracy can be reached for every sequence type that has been included in the training dataset, but a larger number of “junk” scans is needed to reach similar accuracy since the category contains several sequence types.

### Feature Importance

**FIGURE 3:**
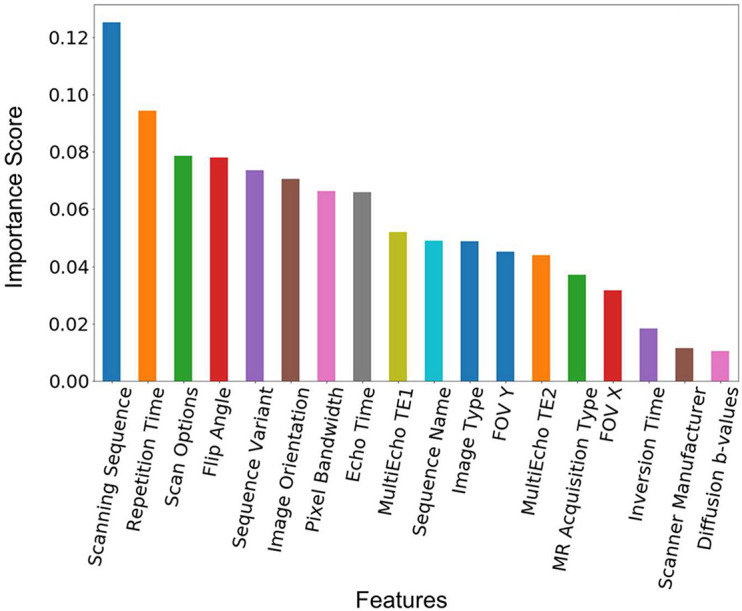
Feature importance score. The feature importance score was extracted from a model built from the training data with 1,200 scans from each of the sequence types 1–7 in Table 2. The features are ranked by the value of the importance score. The corresponding DICOM tags of these features can be seen in Table 3.

The feature importance was extracted from the model built from the training data with 1,200 of each of the sequence types 1–7 in Table 2. Figure 3 shows the ranked feature importance. We can see that (*0018*,*0020*) *Scanning Sequence* ranked the highest among all the selected features, with an importance score >0.10. Other sequence-specific DICOM headers such as (*0018*,*0021*) *Sequence Variant*, (*0018*,*0022*) *Scan Options*, (*0018*,*0024*) *Sequence Name*, *etc*., also have relatively high importance scores. These results are expected since these DICOM headers are designed to carry essential information about the MRI pulse sequence types (Inglis, 2015). The timing parameters such as (*0018*,*0080*) *Repetition Time*, (*0018*,*0081*) *Echo Time*, as well as echo time parameters from Multi-Echo pulse sequences, also have very high importance scores. The three parameters that have relatively low importance scores are the scanner manufacturer, diffusion b-values, and the inversion time. The low ranks of the latter two attributes are apparently because they are only relevant to a small set of sequences in the dataset used in the current study. Nonetheless, they are essential to identify some sequence types such as FLAIR and diffusion weighted images (King et al., 2004). The attribute scanner manufacturer is also a useful feature as some attributes, such as diffusion *b*-values, are encoded in different DICOM headers depending on the scanner manufacturers. Therefore, we kept all the manually selected features listed in Table 3 in the model building and testing processes.

As mentioned above, the training dataset were collected from over 20 image centers by three different research projects. Each of the selected features has many different variations, and covers a wide range of parameter values. For example, for the 7,482 fMRI scans, the TR value ranges from about 1,500 to 2,800 ms, the sequence names have three different variations plus missing or empty fields; for the 9,385 DTI scans, the TR value ranges from about 3,800 to 14,000 ms, the sequence name have 148 different variations. The prediction accuracy is nonetheless very high for all the sequences. We note the sequence name here refers to the DICOM header (*0018*,*0024*), which is generated automatically by the vendor software. Another DICOM header (*0008*,*103E*), *Series Description*, which is an explicit input from the technicians, is usually used to identify the sequence type. The latter has been excluded in our feature list because we have seen too much arbitrariness from this field.

### Classification Confidence

**FIGURE 4:**
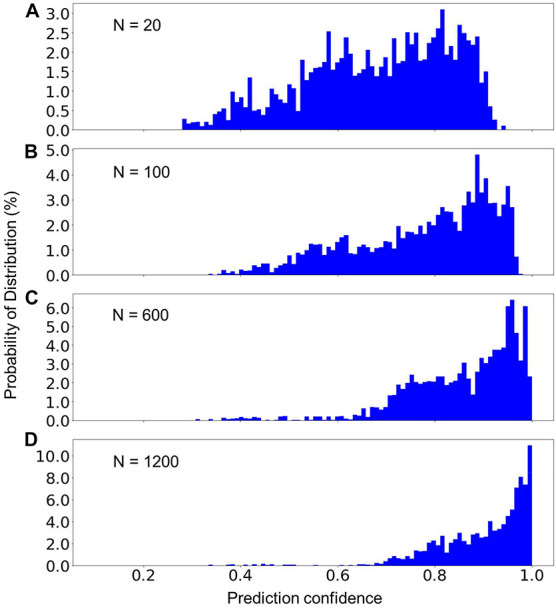
The percent distribution of the prediction confidence of the random forest model built from different sizes of the training datasets. From **(A–D)**, the training datasets consisted of 20, 100, 600, and 1,200 scans from each of the sequence types 1–7 listed in Table 2. The data are collected from 20 separate computations.

Although the average prediction accuracy of the built model can be very high, it is helpful to know the confidence of a prediction in order to determine whether another, typically manual verification step, is needed. Figure 4 presents the distribution of the prediction confidence of the testing dataset, using the model built from three different sizes of the training data. We can see that with the increase of the size of the training dataset, the prediction confidences clearly shifted toward 1.0. As shown in Figure 4D, only a very small fraction (14%) of the training data has a prediction confidence less than 0.8. The predictions with low confidence are typically from scans that are missing relevant DICOM headers. The overall prediction confidence is expected to continue increasing as the size of the training dataset further increases. However, the size of the training dataset in Figure 4D is already very close to the population, 1458, of sequence 2D FLAIR (see Table 2). Thus, we did not further increase the training dataset in the present study.

### Prediction of Unknown Classes

**FIGURE 5:**
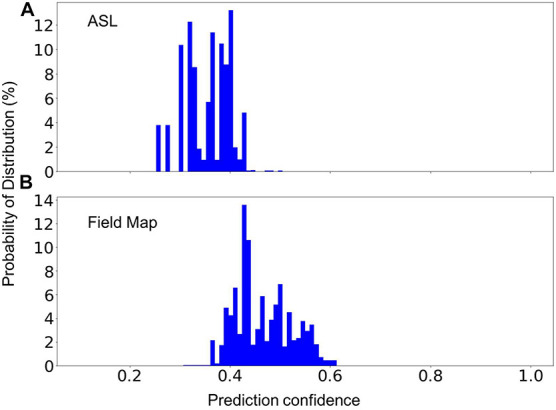
The percent distribution of the classification confidence on predicting two unknown classes, **(A)** Arterial Spin Labeling (ASL), and **(B)** Field Map scans. The random forest models are built from 1,200 scans from each of the sequence types 1–7 listed in Table 2. The data are collected from 20 separate computations.

In MRI data collecting practices, it is common to see sequences that do not exist in the training dataset. Therefore, a machine learning model can only be used in production if it can recognize the unknown sequences and report to the researcher that these sequences cannot be processed automatically. To test the model’s capability to recognize unknown classes, we used the random forest model trained with 1,200 scans from each of MRI sequences 1–7 (known classes) to predict the scans of sequence types 8 and 9 (unknown classes) listed in Table 2. Figure 5 shows the distribution of the prediction confidence. The prediction confidence is mostly distributed in the range of 0.25–0.45 for scans of sequence type 8, and in the range of 0.4–0.6 for scans of sequence type 9. Comparing to the predictions of the known classes shown in Figure 4D, where the prediction confidences concentrated toward 1, we can see that the prediction confidence can be used to detect and report the MRI scans that do not belong to any of the known sequence types.

**FIGURE 6:**
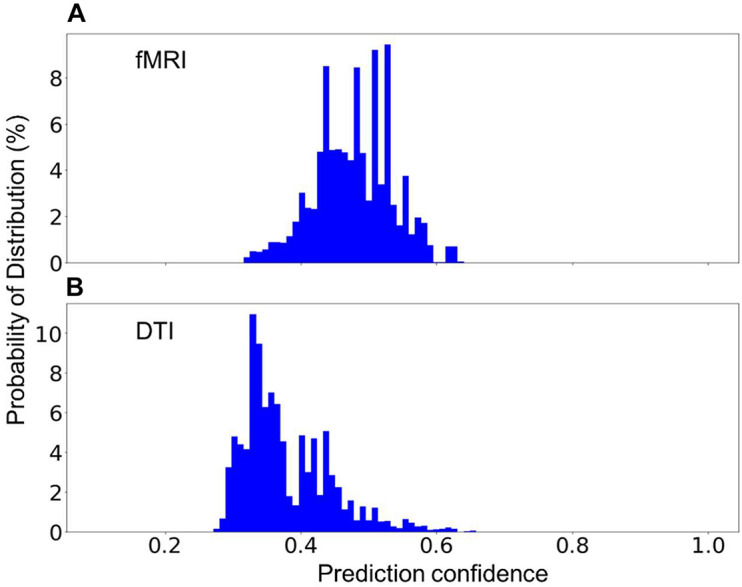
The percent distribution of the classification confidence on predicting two unknown classes, **(A)** fMRI, and **(B)** DTI scans. **(A)** The random forest models are built from 1,200 scans from each of the sequence types, 1–5, and 7, listed in Table 2, i.e., without the fMRI scans. **(B)** The random forest models are built from 1,200 scans from each of the sequence types, 1–4, 6, and 7, i.e., without DTI scans. The data are collected from 20 separate computations.

In practice, one might encounter new sequences that have similar parameters as one of those that were used to build the model. For instance, fMRI and DTI sequences could have some identical parameters as they both rely on the echo-planer imaging technique. Two additional models have been built, each excluding the fMRI or the DTI sequences. The excluded sequences were then treated as an unknown sequence for testing. Figure 6 shows the distribution of the prediction confidence. When fMRI was treated as an unknown sequence, 99.5% of the fMRI scans were predicted to be DTI, and 0.5% were predicted to be in junk category. When DTI was treated as an unknown sequence, 70% of the scans were predicted to be fMRI, 27% were predicted to be 2D Flair, and 3% were predicted to be PD/T2 scans. In both cases, the prediction confidence is still mostly distributed in the range of 0.3–0.6, sufficiently lower than the prediction confidences for the known sequences shown in Figure 4D to require further investigation.

## Discussion

Rapid advancements in neuroimaging technologies and data storage technologies have offered new perspectives in studying neuroscience problems (Frégnac, 2017; Landhuis, 2017). One of the biggest challenges that all researchers in the field are facing is to effectively identify/recognize the datasets that are available to explore. We have tried a few methods, including k-means clustering (Lloyd, 1982), association rule learning (Agrawal et al., 1993), and a generalized correspondence analysis method (Beaton et al., 2019), to separate the scans by grouping their imaging parameters, but all these attempts were unsuccessful. There are two possible reasons for this, (a) any value range could be shared by multiple sequences, e.g., the value ranges of TR for 3DT1, PD/T2, and fMRI scans in this dataset are 6.4–2,740, 2,017–16,000, and 3,800–14,000 ms, respectively; and (b) any of the DICOM headers could be missing, e.g., nearly half of the sequences collected by two Brain-CODE study programs missed the DICOM header (0018,0024), *Sequence Name*, as mentioned above. Therefore it is very difficult to set up a predetermined decision path to identify the MRI sequence types.

Here we used a random forest model, which uses all the available features to establish multiple decision paths, and then combine them together to make a final prediction. Using a domain-knowledge guided approach, we selected a rather small set of imaging metadata (Table 3) as the features for the random forest algorithm, yet the trained models show high prediction accuracies and high degrees of repeatability (Figure 2). Because the metadata listed in Table 3 are based on our understanding of the sequences collected by three research programs used in this study, this list might need to be reduced or extended if the model is used to predict scans of other new sequence types in the future. For example, when dealing with high-resolution fMRI scans, the parameter “Pixel Bandwidth” may need to be reconsidered, otherwise low prediction confidence or even mislabeling could be generated. Also, the list of features needs to be re-examined if other data types such as computerized tomography (CT) and positron emission tomography (PET) scans are included.

Extracting the relative feature importance as shown in Figure 3 can be useful in selecting new features or removing unnecessary features when investigating different datasets. It requires specific attention to interpret the feature importance since some of the features could be correlated to each other. For the random forest algorithm, any of the correlated features can be used to build the model. Once one of the features was used, the importance of the other correlated features could be significantly reduced. When interpreting the data, it can lead to the incorrect conclusion that one feature is a stronger predictor than other correlated features (Dormann et al., 2013). When working with DICOM data, however, it is helpful to keep the correlated features since it is common to encounter missing or incorrect values.

This preliminary study highlights the critical role of the size of the training dataset. As expected, the model clearly gained higher prediction confidence when trained with a larger size of training dataset (Figure 4). In practical applications, a threshold such as 0.8 and 0.9 of the classification can be defined and used to flag the image sequences for researchers to verify. Despite the fact that even with the largest training dataset in this study, 14% of the scans were still predicted with a prediction confidence less than 0.8, the trend shown in Figure 4 is nevertheless promising. As more data were added into the training dataset, there would be less and less predictions requiring manual verification. Note since the random forest algorithm determines its prediction by multiple relatively uncorrelated decision paths, correct predictions can still be expected if a subset of the DICOM headers of a scan are missing and/or incorrect, albeit with lower confidence scores.

The built model is not able to predict the type of the sequences that do not exist in the training data set, but it is necessary for the model to be able to flag these sequences for manual classification since it is common to encounter sequences that are unknown to the model. We demonstrate that the prediction confidence can be used to detect and report sequences that are unknown to the model, where a very low prediction confidence would be generated (Figures 5, 6). However, if the parameters of an unknown sequence are highly similar or even identical to that of a known sequence, mislabeling could occur. In such situations, a second model that includes more relevant parameters, such as series description and number of image frames, could be trained to further separate the scans that are predicted as in the same category by the first model.

We utilized Apache Spark and its machine learning library MLlib to accelerate the computations in the present study (Meng et al., 2016), so that each model training and testing process can be completed in a few minutes. The random forest algorithm used in this work, is one of the most popular machine learning algorithm and it has been implemented in many different computer programming languages including Python library scikit-learn (Pedregosa et al., 2011) and R library caret (Kuhn, 2008), and both libraries are widely used in the neuroscience community. The proposed method can be readily used by other research groups. As most image processing software packages require *a priori* knowledge of the types of the MRI sequences to be processed, the current machine learning approach can be used as a pre-processing step for running the image processing software. In addition, it can also be used in data platforms to standardize sequence naming, and to help convert the existing data into a standardized format such as BIDS (Gorgolewski et al., 2016; Niso et al., 2018).

## Conclusion

Inconsistencies in MRI sequence naming have been a known issue in the neuroscience community for a long time. This issue becomes increasingly pressing because of the rapid growth of the volume of data that are mainly driven by technological advancements and data sharing efforts. In this study, we investigated the possibility of using imaging metadata to automatically identify the type of MRI sequences through machine learning approaches. We used MRI data that were collected from multiple institutes and scanners from over 10 years of time. The preliminary results showed that a random forest model can be trained to accurately (>99.9%) predict MRI sequence types. We demonstrated that the prediction confidence can be used to recognize scans that do not belong to any sequence type that are known to the model. This approach has the potential to allow standardizing sequence naming and processing imaging sequences automatically.

Additionally, this study highlights the importance of the size of the training dataset, where the prediction confidence of known classes clearly increases with increasing the size of the training dataset. Efforts are underway to expand the training dataset and implement the current machine learning model into a portable software that can be easily integrated into other image processing software. With the help of the proposed method, an automatic image categorization process that requires minimal human interventions is well expected. The current procedure can readily be adapted for identifying other image data, such as CT, PET, X-ray and ultrasound, in DICOM formats. Such adaption depends on the availability of large amounts of pre-labeled data for training the classifier. In a broader context, the metadata learning approach used here can be expected to play an important role in organizing and managing medical imaging data in the age of big data.

## Data Availability Statement

The data analyzed in this study is subject to the following licenses/restrictions: The original data will be available through Brian-CODE Open Data Release. Requests to access these datasets should be directed to https://www.braincode.ca/content/open-data-releases.

## Ethics Statement

The studies involving human participants were reviewed and approved by all recruitment sites in accordance with the Governance Policy of Ontario Brain Institute as well the Institutional Policies. Written informed consent to participate in this study was provided by the participants’ legal guardian/next of kin.

## Ondri Founding Authors

We would like to acknowledge the ONDRI Founding Authors: Angela Troyer, Anthony E. Lang, Barry Greenberg, Chris Hudson, Dale Corbett, David A. Grimes, David G. Munoz, Douglas P. Munoz, Elizabeth Finger, J. B. Orange, Lorne Zinman, Manuel Montero-Odasso, Maria Carmela Tartaglia, Mario Masellis, Michael Borrie, Michael J. Strong, Morris Freedman, Paula M. McLaughlin, Richard H. Swartz, Robert A. Hegele, Robert Bartha, Sandra E. Black, Sean Symons, Stephen C. Strother, and William E. McIlroy.

## Author Contributions

SL and SCS designed the study. SL and SRA contributed to initial data curation, while SL performed data preparation, data cleaning, designed and conducted ML experiments, and drafted the manuscript. DB helped design ML experiments. SRA, SH, and SCS provided major contributions to manuscript edits. All authors reviewed and approved the final manuscript.

## Author Disclaimer

The opinions, results, and conclusions are those of the authors and no endorsement by the Ontario Brain Institute is intended or should be inferred.

## Conflict of Interest

The authors declare that the research was conducted in the absence of any commercial or financial relationships that could be construed as a potential conflict of interest.

## Publisher’s Note

All claims expressed in this article are solely those of the authors and do not necessarily represent those of their affiliated organizations, or those of the publisher, the editors and the reviewers. Any product that may be evaluated in this article, or claim that may be made by its manufacturer, is not guaranteed or endorsed by the publisher.

## Footnotes

1
https://surfer.nmr.mgh.harvard.edu

2
https://nifti.nimh.nih.gov

3
https://www.dicomstandard.org/current

4
https://github.com/nipy/heudiconv

5
https://github.com/ReproNim/reproin

6
https://bids-specification.readthedocs.io/en/stable/04-modality-specific-files/01-magnetic-resonance-imaging-data.html

7
https://spred.braincode.ca
